# Does genome size drive the pH-related shifts in bacterial biodiversity within forest soils?

**DOI:** 10.3389/fmicb.2026.1808661

**Published:** 2026-04-24

**Authors:** Yifan Xue, Yuxuan Liu, Jian Gao

**Affiliations:** 1 College of Life and Environment Sciences, Minzu University of China, Beijing, China; 2 School of Ecology and Environment, Baotou Teachers' College, Inner Mongolia University of Science and Technology, Baotou, China

**Keywords:** bacterial biodiversity, bacterial genome size, forest ecosystems, fungal factors, geographical N/P limitation, soil pH

Over the last decade, the mechanisms underlying changes in bacterial biodiversity in forest soils based on 16S rRNA sequencing have gained considerable attention in ecological and geographical research (Bahram et al., 2018; Piton et al., 2023). Soil pH plays a pivotal role in the development of soil bacterial biodiversity (Ramoneda et al., 2023; Luan et al., 2023; He et al., 2023). To explore the mechanism underlying the changes in bacterial biodiversity in forest soils with varying pH, researchers have evaluated bacterial 16S diversity. Wang et al. (2023) analyzed the 16S and bacterial metagenomic data from soil samples obtained at 12 forest sites in China and concluded that on a pH gradient, the mismatch between taxonomic and functional diversity may be attributed to changes in genome size. That is, a small community of large-genome taxa could harbor higher functional diversity than a large community of small-genome taxa. Biodiversity in resource-limited conditions with acidic pH environments (commonly found at low latitudes) should be lower than that in resource-abundant conditions with neutral pH environments (commonly found at high latitudes). Yet this proposed decoupling stands in contrast to the theoretical expectation that genome size and biodiversity exhibit a positive correlation under natural ecological conditions (Lynch and Conery, 2003; Joyce, 2002; Pellicer et al., 2018). Wang et al. (2023) demonstrated that changes in genome size cause a decoupling between bacterial taxonomic and functional diversity, which is highly problematic.

Therefore, the question remains: if not genome size, what drives the decoupling of bacterial taxonomic and functional diversity? It is worth noting that this biodiversity is likely shaped by a combination of biotic and abiotic factors-most notably geographic and fungal influences-which play a decisive role (Xu et al., 2020; Nikitin et al., 2022). Several national-scale studies on forest ecology have demonstrated that broad geographic variations are the primary drivers of bacterial diversity, while fungi represent an essential component of forest soil microbial communities (Bahram et al., 2018; Liu et al., 2020; Song and Zhou, 2021). While Wang et al. (2023) provided valuable insights, the influence of geographical and fungal factors was not fully explored. In the present work, we re-analyzed their dataset by incorporating these variables to better understand the mechanisms driving forest soil bacterial biodiversity across pH gradients. Our analysis integrates data from Wang et al. (2023), Zheng et al. (2021), and Du et al. (2020), all of whom utilized identical sampling sites, methods, and experimental conditions. Since the measurements and primary analyses were conducted by the same research group, the datasets are highly comparable. All statistical analyses were performed in IBM SPSS 27.0 (IBM Inc., New York, NY, USA), and line graph plotting was conducted using OriginPro 2022 (OriginLab Corp., Northampton, MA, USA).

## Influence of geographical factors

Forest soil pH in China peaks near 40° N (Zheng and Song, 2022). While Wang et al. (2023) reported a positive linear correlation, our analysis revealed a nonlinear relationship, particularly near neutral pH. Previous studies have also shown that if Wang et al. could add more sampling sites in the north of 40 °N, the unimodal curve could be more clearly observed (Song, 2023). In our study, bacterial biodiversity showed a significant unimodal relationship with latitude (Supplementary Figure S1a). Furthermore, unimodal relationships between bacterial functional diversity, genome size, and carbohydrate-active enzyme gene diversity and latitude were observed through metagenomic annotation and quantification of diversity indices based on richness (Supplementary Figures S1b–d); all plot visualizations were generated in OriginPro 2022 (OriginLab Corp., Northampton, MA, USA). Our work aligns with previous studies showing a unimodal pattern of bacterial biodiversity around 40° N (Liu et al., 2020). Therefore, the interpretation presented by Wang et al. (2023) may be affected by the fact that they only selected three sites north of 40° N, which may have been insufficient to clearly reveal the unimodal pattern. Meanwhile, both bacterial functional diversity and genome size display a significant decreasing—increasing relationship with latitude, also turning near 40° N (Supplementary Figures S1b–d). Bacterial biodiversity is primarily linked to latitude, whereas functional diversity and genome size correlate more closely with pH. Thus, the observed large genomes and high functional diversity in acidic soils likely reflect the prevalence of such soils in climatically favorable low-latitude forests, not a direct stress response, leading to the earlier misinterpretation.

## Influence of fungal factors

Fungi, particularly mycorrhizal fungi, are widely recognized as key drivers of microbial diversity in forest soils of China due to their central role in nutrient cycling and cannot be overlooked (Chen et al., 2022). We found that latitude indirectly affects fungal diversity by positively influencing plant nitrogen and phosphorus reuptake rates (NR/PR), and NR/PR reflects N/P limitation (Du et al., 2020). A possible explanation for this pattern is that when plants are limited by phosphorus, they become more reliant on symbiotic fungi for nutrient supply (Zheng and Song, 2022). This may create a cascading effect on the dependence of the plants on symbiotic fungi, thereby shaping the latitudinal variation pattern of fungal diversity (Du et al., 2020; Zheng and Song, 2022; Wang et al., 2025; Xue et al., 2025). While fungal and bacterial taxonomic diversity are significantly positively correlated (Supplementary Figure S2a), no such correlation exists for functional diversity. Fungal diversity and bacterial carbohydrate-active enzyme gene diversity (Supplementary Figure S2b) and genome size (Supplementary Figure S2c) were analyzed for pairwise correlations via IBM SPSS 27.0 (IBM Inc., New York, NY, USA), with diversity metrics based on richness for all microbial indices. In contrast to the taxonomic pattern, fungal diversity is negatively correlated with the diversity of bacterial carbohydrate-active enzyme genes (Supplementary Figure S2b). This observational pattern is consistent with the dominant role of mycorrhizal fungi in the carbon cycle, where they directly exchange carbon, nitrogen, and phosphorus with plant roots (Chen et al., 2022; Lebreton et al., 2021). Their symbiotic advantage suggests they may occupy key carbon-metabolism niches, potentially contributing to competitive exclusion of bacteria from these functions (Song et al., 2023). However, the nutrients processed and released by fungi into the soil create new ecological niches, which we interpret as consistent with a “trickle-down effect” that promotes bacterial taxonomic diversity and may account for the observed positive correlation (Jacoby and Kopriva, 2019). Notably, fungal diversity shows no linkage to bacterial genome size (Supplementary Figure S2c), suggesting that these putative fungal-driven processes operate largely independently of genome size evolution in bacteria.

## Verification of the structural equation model

**Figure 1:**
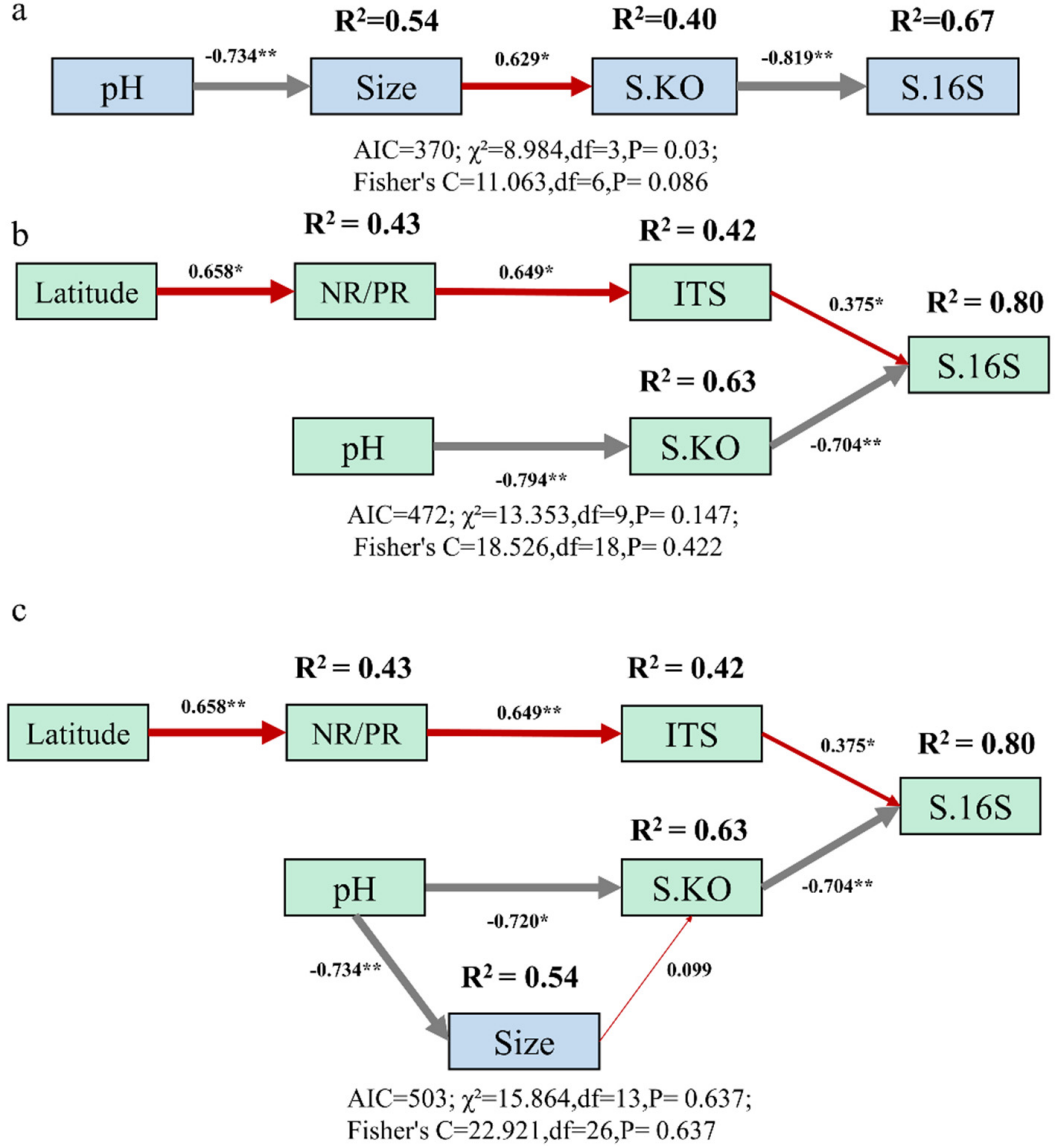
Structural equation models of **(a)** Wang et al. (2023), **(b)** this study, and **(c)** the combination model of the two that describing the effects of multiple drivers on bacterial taxonomic diversity (S.16S). Numbers adjacent to arrows show the effect size of the relationship. Positive and negative relationships between pairwise predictors were differentiated by red and gray color, respectively. The results of AIC, χ^2^, and Fisher's *C*-test are attached below the models. ITS and S.KO refer to fungal taxonomic diversity and bacterial functional diversity, respectively. Symbols on the upper left of the numbers: *p < 0.05; **p < 0.01. (Significance levels for the path coefficients.)

To verify whether our inference is consistent with the actual situation, we used structural equation modeling in R 4.4.1 (packages lavaan v0.6-11 and piecewiseSEM v2.1.0) to examine the ecological processes suggested by (H1) Wang et al. and (H2) this study (Figures 1a, b). The results showed that H2 had a higher degree of explanation for bacterial diversity than H1. Because in the collinearity detection, latitude and pH not only did not show a significant linear correlation (*R*^2^ = 0.213, *P* = 0.131), but the VIF was 1.27, which is less than 5, which indicates a collinear relationship. Therefore, we combined the two models into one for verification and found that the effect size of the relationship between genome size and functional diversity was very small, less than 0.1 (Figure 1c). This confirms our assertion that although soil pH had a significant impact on genome size, genome size had a very small impact on functional diversity, and therefore was not the main driver of bacterial diversity in the context of large-scale forest soil pH gradients in China. Specifically, the ratio of the absolute value of the parameter estimate to the standard error for the pathway from bacterial genome size to functional diversity is approximately 0.336 (Supplementary material S1), which is far below the 1.96 threshold for the 95% confidence interval, indicating that the observed weak effect of this pathway is merely due to random error rather than a true causal relationship. Notably, this pathway is the only one with a non-significant effect among all pathways affecting bacterial taxonomic diversity (S.16S), which effectively excludes systematic error as a potential confounding factor. We recognize that such genome size effects may be scale dependent and context specific, and are not intended to imply they are universally negligible in other ecological systems or spatial scales. In conclusion, the verification results of the structural equation model indicate that H2 was clearly more consistent with the actual situation than H1, as the *p*-values derived from both the chi-square test and Fisher's *C*-test all demonstrated that the fit of H2 was far superior to that of H1, leading to this conclusion, indicating that the variation in bacterial biodiversity in forest soils in China with pH is not driven by genome size, but mainly by geographical and fungal factors.

## Conclusion and suggestions

This study demonstrates that pH-driven variations in forest soil bacterial biodiversity across China are not governed by genome size, as previously suggested, but are primarily influenced by geographical and fungal factors. The earlier conclusion-that the decoupling of bacterial taxonomic and functional diversity stems from changes in genome size-likely overlooked the confounding effects of site heterogeneity and large-scale geographical influences. To enhance the robustness of future research, we propose: (1) accounting for geographical and fungal variables; (2) ensuring the selection of representative sampling sites; and (3) employing transect-based or controlled experiments to better isolate environmental drivers.

## Supplementary material

Supplementary Figure S1 Relationships between latitude and bacterial **(a)** taxonomic diversity (S.16S), **(b)** diversity of carbohydrate-active enzymes genes (S.Cazy), **(c)** functional diversity (S.KO), and **(d)** average genome size. Data from 36 sampling sites were used at 95% confidence intervals. Diversities shown were measured by richness. Bacterial carbohydrate-active enzymes genes were detected based on shotgun metagenome annotated by the database of CAZy. Bacterial functions were determined from the shotgun metagenome, as annotated by the Kyoto Encyclopedia of Genes and Genomes Ontology.

Supplementary Figure S2 Relationships between fungal taxonomic diversity (ITS) and **(a)** bacterial taxonomic diversity (S.16S), **(b)** bacterial diversity of carbohydrate-active enzymes genes (S.Cazy), and **(c)** bacterial average genome size. Data from 12 sampling sites were used at 95% confidence intervals. Diversities shown were measured by richness. Bacterial carbohydrate-active enzymes genes were detected based on shotgun metagenome annotated by the database of CAZy. Bacterial functions were determined from the shotgun metagenome, as annotated by Kyoto Encyclopedia of Genes and Genomes Ontology.
