# Supplementary material for: Does genome size drive the pH-related shifts in bacterial biodiversity within forest soils?
Source: Front Microbiol. 2026 Apr 24;17:1808661. doi: 10.3389/fmicb.2026.1808661 (PMC13153058; doi:10.3389/fmicb.2026.1808661)
Supplement: Supplementary file 1 [file Table_1.docx]

**A:**Structural Equation Model of mydata_psem

Call:

S.16S ~ S.KO

S.KO ~ Size

Size ~ pH

AIC

369.639

---

Tests of directed separation:

Independ.Claim Test.Type DF Crit.Value P.Value

S.KO ~ pH + ... coef 9 -2.4261 0.0382 *

S.16S ~ pH + ... coef 9 1.3115 0.2221

S.16S ~ Size + ... coef 8 0.7649 0.4663

--

Global goodness-of-fit:

Chi-Squared = 8.984 with P-value = 0.03 and on 3 degrees of freedom

Fisher's C = 11.063 with P-value = 0.086 and on 6 degrees of freedom

---

Coefficients:

Response Predictor Estimate Std.Error DF Crit.Value P.Value Std.Estimate

S.16S S.KO -2.5986 0.5761 10 -4.5108 0.0011 -0.8188 **

S.KO Size 125.6367 49.1654 10 2.5554 0.0286 0.6285 *

Size pH -0.7261 0.2124 10 -3.4183 0.0066 -0.7341 **

Signif. codes: 0 '***' 0.001 '**' 0.01 '*' 0.05

---

Individual R-squared:

Response method R.squared

S.16S none 0.67

S.KO none 0.40

Size none 0.54

**B:**Structural Equation Model of mydata_psem

Call:

S.16S ~ S.KO + ITS

S.KO ~ pH

ITS ~ NP

NP ~ Latitude

AIC

471.795

---

Tests of directed separation:

Independ.Claim Test.Type DF Crit.Value P.Value

NP ~ pH + ... coef 9 2.1627 0.0588

S.16S ~ pH + ... coef 8 0.4029 0.6975

ITS ~ pH + ... coef 9 0.4210 0.6836

S.KO ~ Latitude + ... coef 9 -0.3736 0.7173

S.16S ~ Latitude + ... coef 8 0.7497 0.4749

ITS ~ Latitude + ... coef 9 -2.4175 0.0388 *

NP ~ S.KO + ... coef 8 0.5950 0.5683

ITS ~ S.KO + ... coef 8 0.5428 0.6021

S.16S ~ NP + ... coef 7 -0.3331 0.7488

--

Global goodness-of-fit:

Chi-Squared = 13.353 with P-value = 0.147 and on 9 degrees of freedom

Fisher's C = 18.526 with P-value = 0.422 and on 18 degrees of freedom

---

Coefficients:

Response Predictor Estimate Std.Error DF Crit.Value P.Value Std.Estimate

S.16S S.KO -2.2347 0.4998 9 -4.4717 0.0016 -0.7042 **

S.16S ITS 4.2221 1.7747 9 2.3791 0.0413 0.3747 *

S.KO pH -156.9055 38.0535 10 -4.1233 0.0021 -0.7935 **

ITS NP 82.3186 30.5346 10 2.6959 0.0225 0.6488 *

NP Latitude 0.0286 0.0103 10 2.7657 0.0199 0.6583 *

Signif. codes: 0 '***' 0.001 '**' 0.01 '*' 0.05

---

Individual R-squared:

Response method R.squared

S.16S none 0.80

S.KO none 0.63

ITS none 0.42

NP none 0.43

**C:**Structural Equation Model of mydata_psem

Call:

S.16S ~ S.KO + ITS

S.KO ~ pH + Size

ITS ~ NP

Size ~ pH

NP ~ Latitude

AIC

503.114

---

Tests of directed separation:

Independ.Claim Test.Type DF Crit.Value P.Value

NP ~ pH + ... coef 9 2.1627 0.0588

S.16S ~ pH + ... coef 8 0.4029 0.6975

ITS ~ pH + ... coef 9 0.4210 0.6836

S.KO ~ Latitude + ... coef 8 -0.3157 0.7603

Size ~ Latitude + ... coef 9 -0.3867 0.7080

S.16S ~ Latitude + ... coef 8 0.7497 0.4749

ITS ~ Latitude + ... coef 9 -2.4175 0.0388 *

NP ~ S.KO + ... coef 7 0.5196 0.6193

ITS ~ S.KO + ... coef 7 0.4296 0.6804

NP ~ Size + ... coef 8 0.5033 0.6283

S.16S ~ Size + ... coef 7 0.1606 0.8770

ITS ~ Size + ... coef 8 1.3366 0.2181

S.16S ~ NP + ... coef 7 -0.3331 0.7488

--

Global goodness-of-fit:

Chi-Squared = 15.864 with P-value = 0.257 and on 13 degrees of freedom

Fisher's C = 22.921 with P-value = 0.637 and on 26 degrees of freedom

---

Coefficients:

Response Predictor Estimate Std.Error DF Crit.Value P.Value Std.Estimate

S.16S S.KO -2.2347 0.4998 9 -4.4717 0.0016 -0.7042 **

S.16S ITS 4.2221 1.7747 9 2.3791 0.0413 0.3747 *

S.KO pH -142.4141 58.7002 9 -2.4261 0.0382 -0.7202 *

S.KO Size 19.9565 59.3399 9 0.3363 0.7443 0.0998

ITS NP 82.3186 30.5346 10 2.6959 0.0225 0.6488 *

Size pH -0.7261 0.2124 10 -3.4183 0.0066 -0.7341 **

NP Latitude 0.0286 0.0103 10 2.7657 0.0199 0.6583 *

Signif. codes: 0 '***' 0.001 '**' 0.01 '*' 0.05

---

Individual R-squared:

Response method R.squared

S.16S none 0.80

S.KO none 0.63

ITS none 0.42

Size none 0.54

NP none 0.43

**Appendix：The original C**

Structural Equation Model of mydata_psem

Call:

S.16S ~ S.KO + ITS

S.KO ~ pH + Size

ITS ~ NP + pH

Size ~ pH

NP ~ Latitude

AIC

504.880

---

Tests of directed separation:

Independ.Claim Test.Type DF Crit.Value P.Value

NP ~ pH + ... coef 9 2.1627 0.0588

S.16S ~ pH + ... coef 8 0.4029 0.6975

S.KO ~ Latitude + ... coef 8 -0.3157 0.7603

Size ~ Latitude + ... coef 9 -0.3867 0.7080

S.16S ~ Latitude + ... coef 8 0.7497 0.4749

ITS ~ Latitude + ... coef 8 -2.3231 0.0487 *

NP ~ S.KO + ... coef 7 0.5196 0.6193

ITS ~ S.KO + ... coef 7 0.4296 0.6804

NP ~ Size + ... coef 8 0.5033 0.6283

S.16S ~ Size + ... coef 7 0.1606 0.8770

ITS ~ Size + ... coef 8 1.3366 0.2181

S.16S ~ NP + ... coef 7 -0.3331 0.7488

--

Global goodness-of-fit:

Chi-Squared = 15.63 with P-value = 0.209 and on 12 degrees of freedom

Fisher's C = 21.705 with P-value = 0.597 and on 24 degrees of freedom

---

Coefficients:

Response Predictor Estimate Std.Error DF Crit.Value P.Value Std.Estimate

S.16S S.KO -2.2347 0.4998 9 -4.4717 0.0016 -0.7042 **

S.16S ITS 4.2221 1.7747 9 2.3791 0.0413 0.3747 *

S.KO pH -142.4141 58.7002 9 -2.4261 0.0382 -0.7202 *

S.KO Size 19.9565 59.3399 9 0.3363 0.7443 0.0998

ITS NP 69.3776 44.2800 9 1.5668 0.1516 0.5468

ITS pH 8.1815 19.4324 9 0.4210 0.6836 0.1469

Size pH -0.7261 0.2124 10 -3.4183 0.0066 -0.7341 **

NP Latitude 0.0286 0.0103 10 2.7657 0.0199 0.6583 *

Signif. codes: 0 '***' 0.001 '**' 0.01 '*' 0.05

---

Individual R-squared:

Response method R.squared

S.16S none 0.80

S.KO none 0.63

ITS none 0.43

Size none 0.54

NP none 0.43
