# Supplementary figures and images for: Does genome size drive the pH-related shifts in bacterial biodiversity within forest soils?
Source: Front Microbiol. 2026 Apr 24;17:1808661. doi: 10.3389/fmicb.2026.1808661 (PMC13153058; doi:10.3389/fmicb.2026.1808661)

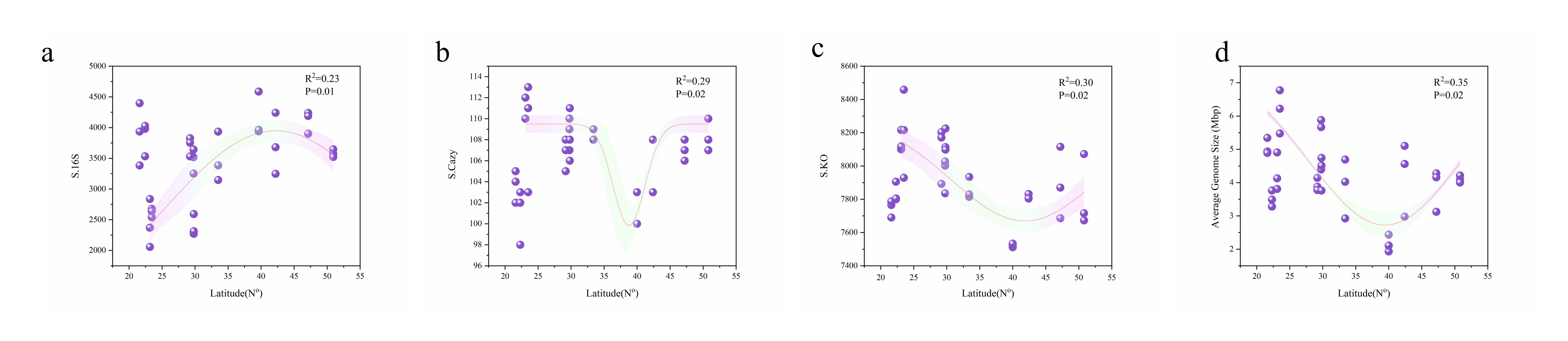

Supplement: Supplementary Figure S1 — Relationships between latitude and bacterial (a) taxonomic diversity (S.16S), (b) diversity of carbohydrate-active enzymes genes (S.Cazy), (c) functional diversity (S.KO), and (d) average genome size. Data from 36 sampling sites were used at 95% confidence intervals. Diversities shown were measured by richness. Bacterial carbohydrate-active enzymes genes were detected based on shotgun metagenome annotated by the database of CAZy. Bacterial functions were determined from the shotgun metagenome, as annotated by the Kyoto Encyclopedia of Genes and Genomes Ontology. [file Image_1.png]

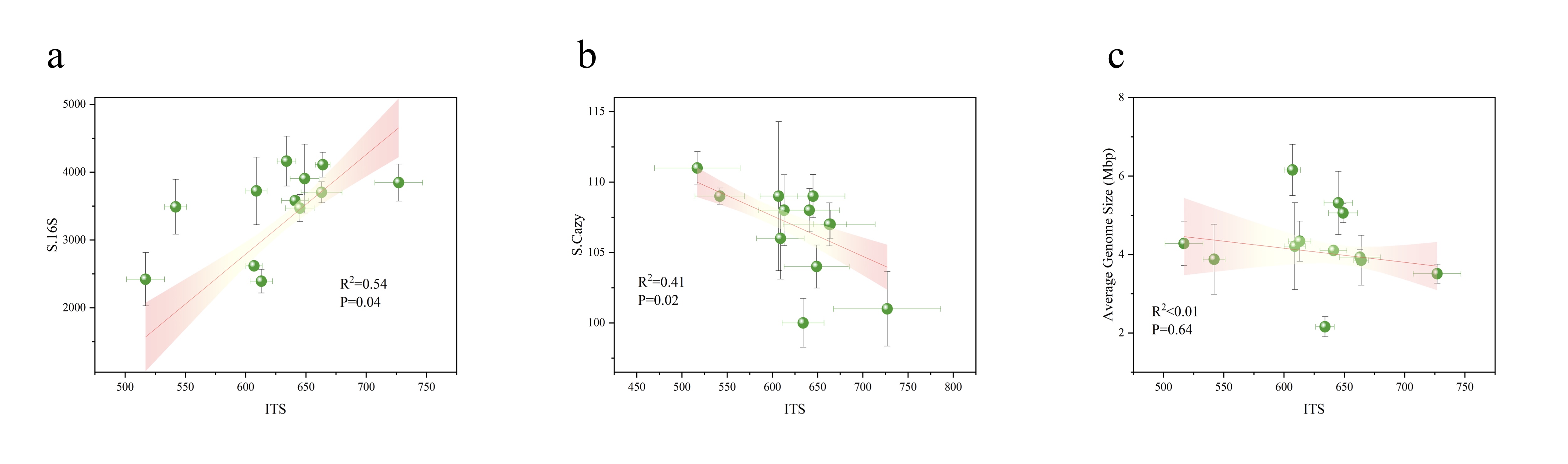

Supplement: Supplementary Figure S2 — Relationships between fungal taxonomic diversity (ITS) and (a) bacterial taxonomic diversity (S.16S), (b) bacterial diversity of carbohydrate-active enzymes genes (S.Cazy), and (c) bacterial average genome size. Data from 12 sampling sites were used at 95% confidence intervals. Diversities shown were measured by richness. Bacterial carbohydrate-active enzymes genes were detected based on shotgun metagenome annotated by the database of CAZy. Bacterial functions were determined from the shotgun metagenome, as annotated by Kyoto Encyclopedia of Genes and Genomes Ontology. [file Image_2.jpeg]
